# *Bacillus amyloliquefaciens* Ameliorates H_2_O_2_-Induced Oxidative Damage by Regulating Transporters, Tight Junctions, and Apoptosis Gene Expression in Cell Line IPEC-1

**DOI:** 10.1007/s12602-019-09538-5

**Published:** 2019-03-20

**Authors:** Yanping Wu, Han Xu, Xuefang Cao, Rongrong Liu, Li Tang, Zhonghua Zeng, Weifen Li

**Affiliations:** Key Laboratory of Molecular Animal Nutrition, Ministry of Education, Institute of Feed Science, College of Animal Science, Zhejiang University, Hangzhou, 310058 China

**Keywords:** Ba, H_2_O_2_, Oxidative stress, mRNA expression

## Abstract

Probiotics have always been considered as a supplementary therapy for many diseases especially gut disorders. The absorption and barrier function of the gut play a vital role in the maintenance of body homeostasis. This study was to investigate the protective effects of *Bacillus amyloliquefaciens* SC06 (Ba) on H_2_O_2_-induced oxidative stress on intestinal porcine epithelial cells (IPEC-1) based on the level of gene expression. We demonstrated that Ba was a safe probiotic strain in the first place. Results showed that treatment with H_2_O_2_ significantly increased the mRNA expression of absorptive transporters glucose transporter 2 (GLUT2), Ala/Ser/Cys/Thr transporter 1 (ASCT1), and ASCT2 compared with the control group. Meanwhile, oxidative stress induced a significant improvement in the mRNA expression of occludin (OCLN) and caspase-3, and remarkably inhibited the expression of L-type amino acid transporter 1 (LAT1) or B cell lymphoma-2 (Bcl-2), respectively. Pretreatment with Ba dramatically reversed the disturbance induced by oxidative stress on the mRNA expression of ASCT1, ASCT2, and OCLN, which also significantly prevented H_2_O_2_-inhibited LAT1 and Bcl-2 mRNA expression. However, Ba failed to exert any significant protective effect on GLUT2 and caspase-3 mRNA expression. We concluded that pretreatment with Ba could alleviate the damage caused by oxidative stress to a certain extent and conferred a protective effect to the intestine.

## Introduction

Animal intestine contains a series of complex commensal and antagonistic bacteria to maintain a balanced environment. In addition, intestinal epithelia also have digestive, absorptive, and defense-barrier functions. The gastrointestinal tract can secrete different kinds of enzymes to decompose foods into micronutrients which could be further assimilated by epithelial cells. Notably, absorption means transporting substrates, including carbohydrates, amino acids or peptides, and fatty acids. These substrates are absorbed into cells by transporters from the intestinal lumen across the brush-border membrane, and enter the blood or lymphatic system subsequently. Intestinal sugar transporters like glucose transporter 2 (GLUT2) and sodium glucose cotransporter 1 (SGLT1) are responsible for transporting the monosaccharides like glucose, galactose, and fructose from the intestinal lumen to the blood [1]. Besides, other major intestinal transporters like neutral amino acid transporter ASCT1 [2] and fatty acid transporter fatty acid translocase (FAT/CD36) [3] influence the absorption of some amino acids and fatty acids. Healthy intestinal mucosal barrier is a key factor in determining whether a disease occurs; therefore, the loss of barrier integrity increases the translocation of bacterial antigens and stimulates inflammation in the gut. An important component of the intestinal barrier is the intercellular junctional complex called tight junctions (TJs): they seal the paracellular space between epithelial cells to protect cells against pathogens [4]. For example, zonula occludens-1 (ZO-1) and claudin-1 (CLDN-1) play central roles in improving the intestinal barrier function. Another vital factor of intestinal integrity is the degree of cell apoptosis. Apoptosis is a normal but an important process that is responsible for maintenance of the cellular balance between proliferation and death; nevertheless, excessive apoptosis destroys intestinal physiological functions, which can bring about celiac disease such as ulcerative colitis [5].

Oxidative stress is considered to be an imbalance between production of free radicals and reactive metabolites called reactive oxygen species (ROS) [6], which leads to the damage of host cells and thus involves a wide spectrum of diseases including chronic inflammation. H_2_O_2_ is a representative of ROS because of its capability of diffusing throughout the mitochondria and across cell membranes, causing many types of cellular injury [7].

Probiotics are live microorganisms that, when ingested in adequate amounts, provide health benefits to the host. More recently, studies have shown that some beneficial effects of probiotic application include regulation of gut microbiota [8], enhancement of the mucosal barrier [9], stimulation of the immune system [10], and protection of epithelia [11]. Our team has previously shown that *Bacillus amyloliquefaciens* SC06 (Ba) is beneficial to intestinal tract of piglets, which also has a great antioxidant effect on IPEC-1 cells by regulating the Nrf2/Keap1 pathway [12, 13], but effects of oxidative stress on intestinal structure and absorptive function were not completely understood. In the current study, we hypothesize that the specific strain Ba might be able to alleviate the effect of H_2_O_2_-induced oxidative damage on absorption, barrier function, and apoptosis of IPEC-1 cells. We determined the mRNA expression of different transporters and proteins concerning barrier and apoptosis.

## Materials and Methods

### IPEC-1 Cell Culture Conditions

The porcine epithelial cell line was kindly given by the Animal Husbandry and Veterinary Research Institute of ZAAS (Zhejiang Academy of Agricultural Sciences). Cells were cultured in Dulbecco’s modified Eagle medium (DMEM/Ham’s F12 (1:1)) supplemented with 10% heat-inactivated fetal bovine serum (FBS) and 1% antibiotic mixture (100 U/mL penicillin and 100 μg/mL streptomycin), grown at 37 °C and 5% CO_2_ in an incubator. Fresh medium was regularly replaced every 48 h; when cells were 80% confluent, the medium was abandoned and cells were washed twice with phosphate-buffered saline (PBS), and trypsinized with 0.25% trypsin solution containing 0.02% EDTA. After centrifugation, the supernatant was discarded; finally, cells were cultured with a fresh medium.

### Bacterial Preparation

The strain Ba was isolated from soil and preserved in China Center for Type Culture Collection. Ba was cultured in LB media. We determined bacterial growth curves by measuring colony forming units (cfu) per milliliter. Bacteria were quantified per milliliter per specific optical density (OD) at 600 nm. Briefly, 200 μL of bacterial glycerol stock was inoculated into 5 mL broth and placed on a shaker incubator from morning until evening to establish the primary culture, and then 2 mL of primary culture was inoculated into 200 mL of broth to establish a secondary culture. The next morning, bacterial cells were harvested from the broth by centrifugation (5000×*g*, 5 min, 4 °C), washed twice with sterile PBS, and resuspended with PBS. Twice echelon dilution was used in this experiment to calculate the required concentration.

### Exposure of IPEC-1 Cells to H_2_O_2_

Cells were routinely seeded at a density of 5 × 10^5^ cfu/mL in 12-well plates (1 mL of cell solution/well). The stock solution of H_2_O_2_ was made in sterile double-distilled water at the concentration of 5 mmol/L. Before treatment, the confluent monolayers of IPEC-1 cells were washed twice with PBS , added with 100μL H_2_O_2_ and then maintained the volume of every well at 1 mL, which meant that cells were apically treated with 500 μM H_2_O_2_. Treatments were divided into four groups as follows: group in blank control (CK); group in probiotic treatment or pretreatment was incubated for 3 h with Ba (10^8^cfu/mL) (Ba), then washed with PBS; probiotic pretreatment and oxidative stress groups were added with 500 μM H_2_O_2_ and cultivated for 12 h (H_2_O_2_, Ba+H_2_O_2_). Each group was tested in triplicate. After 15 h, cells were washed twice with PBS. One milliliter of RNAiso solution was added in every well to lyse cells completely for quantitative PCR (Q-PCR) experiment.

### Determination of Cell Lactate Dehydrogenase

A total of 5 × 10^5^ IPEC-1 cells/well was seeded in 12-well microplates and separated into the following groups, each in triplicate: control group was without any treatment but the medium for 1.5 h, 3 h, and 6 h respectively. The positive group was treated with 1 mL PBS containing 1% Triton X-100 for 15 min before harvesting cells. Probiotic groups P1, P2, and P3 were given apically different concentrations of probiotics (5 × 10^7^, 1 × 10^8^, 2 × 10^8^ cfu/mL) in cell medium and incubated for 1.5 h, 3 h, and 6 h, respectively, in conditions of 37 °C and 5% CO_2_. After treatment, cell culture supernatants were collected by centrifugation at 3500 rpm for 10 min and stored at − 80 °C to be used for determination of lactate dehydrogenase (LDH).

### Quantitative Real-time PCR

Extraction of total RNA and reverse transcription were performed according to kit instructions (TaKaRa). PCR primer sequences for the pig genes were designed and selected by Primer 5.0 and Oligo 7.0 software as presented in Table 1. Glyceraldehyde-3-phosphate dehydrogenase (GAPDH) as housekeeping gene was used to normalize target gene transcript levels. Real-time PCR was performed according to Du et al. [13]. The 2^−ΔΔCt^ method was used to estimate messenger RNA (mRNA) abundance. Relative gene expression levels were normalized by eukaryotic reference gene GAPDH.

**Table 1:** Primer sequences and amplicon length of PCR products

Gene symbol	Gene name	GenBank accession no.	Primer sequence (5′–3′)	Product size (bp)
GAPDH	Glyceraldehyde-3-phosphate dehydrogenase	NM_001206359.1	F: GGTCGGAGTGAACGGATTT	245
			R: ATTTGATGTTGGCGGGAT	
GLUT2	Glucose transporter 2	NM_001097417.1	F: AGGCATATCAGGACTCTACT	77
			R: ACTTGGTTGGAGCAATCT	
PepT1	Peptide transporter 2	NM_214347.1	F: CAACATCATCGTGCTTATC	171
			R: TCGTCCATATCAAACTGAG	
ASCT1	Ala/Ser/Cys/Thr transporter 1	XM_013996100.2	F: AATGGTGTAGACAAGAGGAT	228
			R: CAGGATAATGGCGATGGT	
ASCT2	Ala/Ser/Cys/Thr transporter 2	XM_003355984.4	F: TGGTCTCCTGGATCATGTGGT	203
			R: GAAGCGGTAGGGGTTTTTGC	
EAAC1	Excitatory amino acid carrier 1	NM_001164649.1	F: ATCGTTCAAATCATTATGTG	167
			R: TATATCAGTGGCAGAATAAC	
LAT1	L-type amino acid transporter 1	NM_001110421.1	F: GGAACTGGGCACCACCATTA	161
			R: GCACCACGTAGTTGGCAAAG	
FAT/CD36	Fatty acid translocase	NM_001044622.1	F: AAGAGCATAGCACTTACTT	150
			R:AGAGGATAGGCACGATAT	
OCLN	Occludin	NM_001163647.2	F:GACTCTACGTGGATCAATA	155
			R:CGACTTGTCATAGTGGTA	
ZO-1	Zonula occludens-1	XM_003121673.1	F:GCCTCCTGAGTTTGATAGTGG	287
			R:CTCGGCAGACCTTGAAATAGA	
Bcl-2	B cell lymphoma-2	NM_214285	F:ACCTGAATGACCACCTAGAGC	180
			R:TCCGACTGAAGAGCGAAC	
Cas-3	Caspase-3	NM_214131	F:GTGGGACTGAAGATGACA	190
			R: ACCCGAGTAAGAATGTG	

*F* forward, *R* reverse

### Data Analysis

All data were presented as the mean ± SD. Graphs were performed using GraphPad Prism version 7.0. The significance of differences between groups was determined using one-way analysis of variance (ANOVA) and *P* < 0.05 was considered statistically significant.

## Results

### Different Concentrations of Probiotic Ba Do Not Harm IPEC-1 Cells

**Fig. 1:**
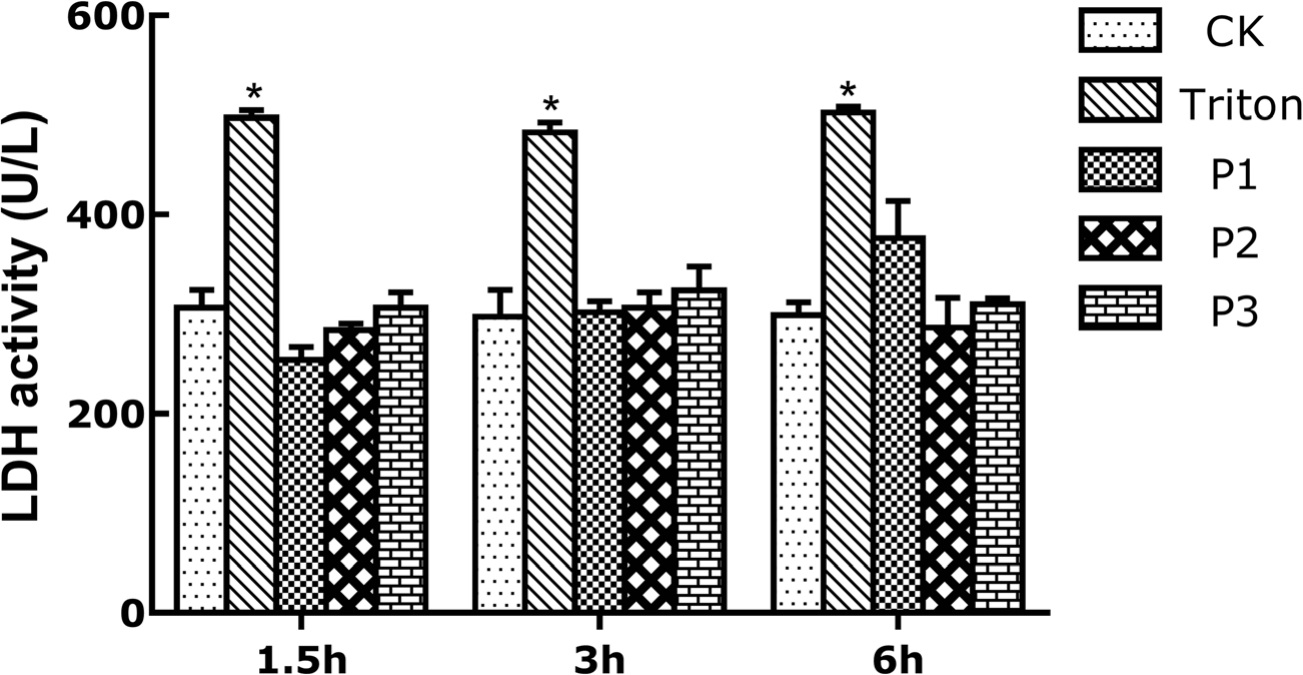
Different concentrations of probiotic Ba do not harm IPEC-1 cells. Data are expressed as mean ± SD. CK, control group; Triton, 1% Triton treatment group; P1, 5 × 10^7^ cfu/mL Ba treatment group; P2, 1 × 10^8^ cfu/mL Ba treatment group; P3, 2 × 10^8^ cfu/mL Ba treatment group. LDH activity was measured after 1.5 h, 3 h, and 6 h in every group. Only 1% triton significantly increased LDH activity (*P < 0.05 versus control and probiotic groups)

The probiotic was non-toxic to the epithelial cells, as shown in Fig. 1. Conversely, cell LDH activity in positive groups was significantly higher (*P* < 0.05) than untreated controls in every time point, which suggested that 1% of Triton X-100 damaged cytomembrane and spilled intracellular LDH. However, compared to control group, no significant change was observed in different doses of probiotic treatment, but it was lower than that in positive groups (*P* < 0.05). These data imply that Ba is not harmful to cells.

### Effects of Ba on Oxidative Stress-Induced Epithelial Absorptive Activity

**Fig. 2:**
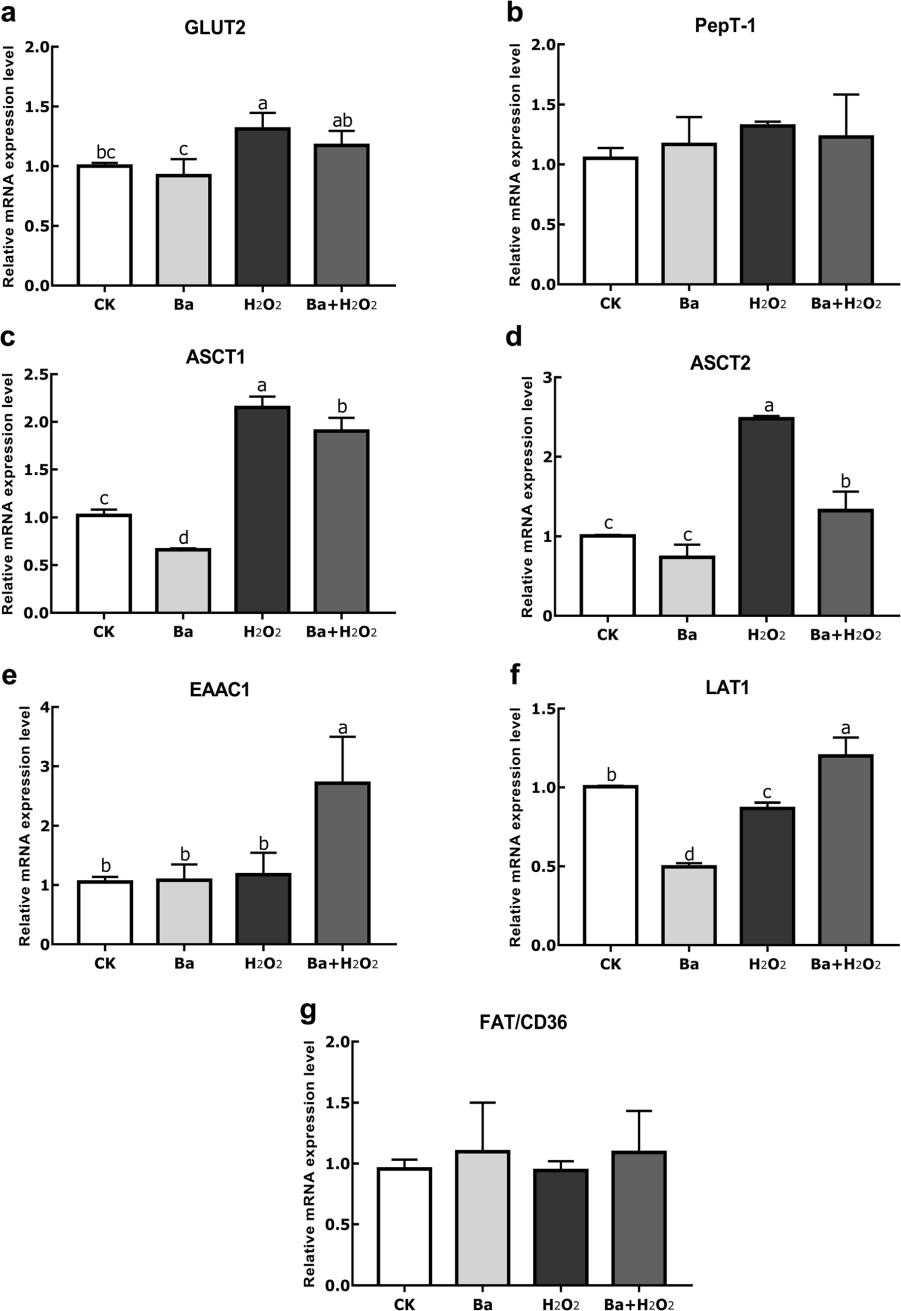
Probiotics and H_2_O_2_ regulated different intestinal epithelial absorptive transporters. Data are expressed as mean ± SD. Bars with different letters are significantly different (*P* < 0.05)

We performed the following studies with the bacterial concentration of 10^8^ cfu/mL. Figure 2 showed higher (*P* < 0.05) GLUT2, ASCT1, and ASCT2 mRNA levels in H_2_O_2_-treated cells than in control group. No significance was detected about GLUT2 mRNA expression in the Ba pretreatment group compared to H_2_O_2_ group, but H_2_O_2_-induced increase in ASCT1 and ASCT2 expression was significantly suppressed by Ba treatment (*P* < 0.05). However, pretreatment with Ba was found to cause a remarkable increase of excitatory amino acid carrier 1 (EAAC1) expression. In contrast, H_2_O_2_ caused a small but significant decrease (*P* < 0.05) in L-type amino acid transporter 1 (LAT1) mRNA expression, while Ba pretreatment markedly reversed the expression of LAT1 (*P* < 0.05). There was no difference in the mRNA expression of peptide transporter 1 (PepT-1) and FAT/CD36 between groups (*P* > 0.05).

### Effects of Ba on Oxidative Stress-Induced Epithelial Barrier Function

**Fig. 3:**
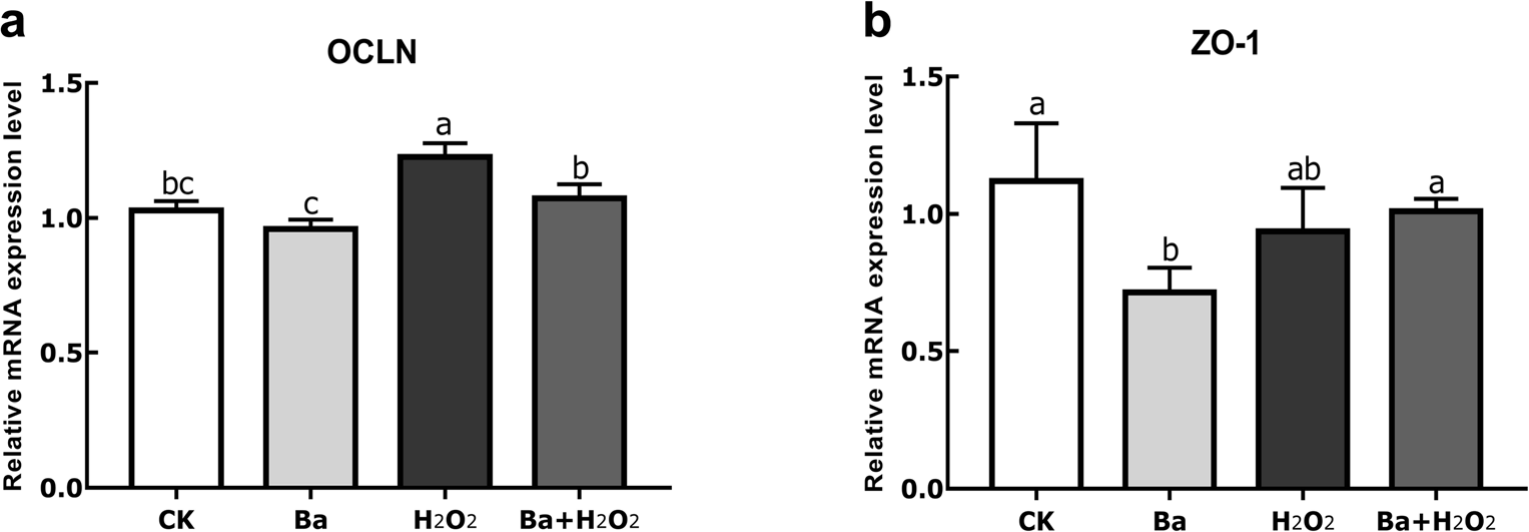
Probiotics and H_2_O_2_ regulated intestinal epithelial TJs. Data are expressed as mean ± SD. Bars with different letters are significantly different (*P* < 0.05)

We analyzed the gene expression of selected tight junctional proteins, as summarized in Fig. 3. Similarly, OCLN mRNA expression in the H_2_O_2_ group was significantly increased compared to control group (*P* < 0.05), whereas Ba pretreatment decreased its mRNA levels (*P* < 0.05). However, there was no change in H_2_O_2_-induced ZO-1 expression when compared to control and Ba pretreatment groups (*P* > 0.05).

### Effects of Ba on Oxidative Stress-Induced Intestinal Cell Apoptosis

**Fig. 4:**
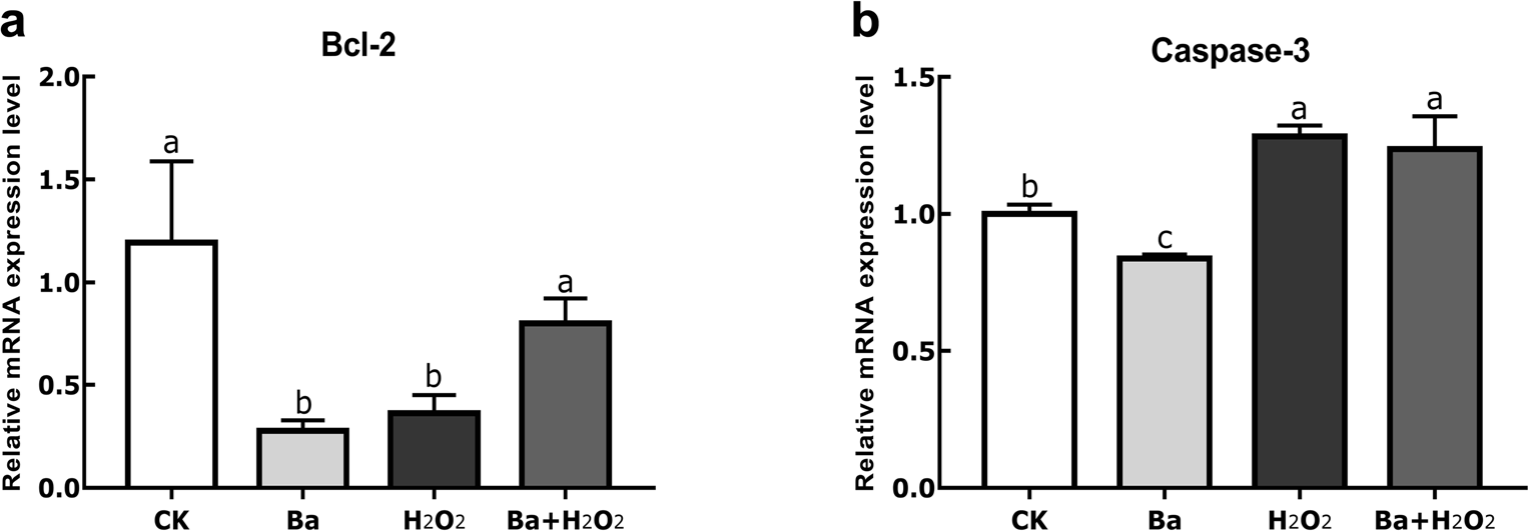
Probiotics and H_2_O_2_ regulated intestinal epithelial apoptosis genes. Data are expressed as mean ± SD. Bars with different letters are significantly different (*P* < 0.05)

To finally assess whether oxidative stress induced intestinal morphology changes and whether Ba could restore it, we determined the mRNA expression of related apoptosis gene (Fig. 4). B cell lymphoma-2 (Bcl-2) levels in H_2_O_2_ group were significantly lower than control (*P* < 0.05), and this effect was completely prevented if cells were pretreated with probiotics (*P* < 0.05). On the contrary, compared to the control group, oxidative stress induced a higher caspase-3 gene expression (*P* < 0.05), but Ba failed to exert a significantly protective effect (*P* > 0.05).

## Discussion

The effect of probiotic bacteria on gastrointestinal digestion and absorption is incompletely understood. Our findings demonstrate the effect of Ba is non-toxic. In previous studies, our team has shown that Ba (1 × 10^8^ cfu/mL) facilitated polarization of M1 macrophages and enhanced its phagocytic capacity in vitro [14]. Moreover, this probiotic strain could induce autophagy-related signaling pathway and improve growth performance of piglets [15, 16]. Different strains of *Bacillus amyloliquefaciens* have been shown to not only improve growth performance, increase the ratio of villus height to crypt depth, and ameliorate cecal microflora in broilers [17, 18], but also regulate cytokine and strengthen host immune status in fish [19].

Generally speaking, oxidative stress can destroy host antioxidant system and maintenance of the cellular balance between proliferation and death. The specific physiological role of transporters expressed in epithelial cells is involved in glucose, protein, and lipid homeostasis. GLUT2 is present in the basolateral membrane of absorptive epithelial cells and is able to transport glucose and fructose and so on [20, 21]. PepT-1 is primarily expressed in the brush-border membranes of the small intestine with the aim of transporting and absorbing di/tripeptides from diet [22]; in addition, neutral amino acid transporters LAT1, ASCT1, and ASCT2 and acid transporter EAAC1 extensively exist in the small intestine. In this study, the mRNA expression of GLUT2, ASCT1, and ASCT2 was generally induced by H_2_O_2_, which was in agreement with the result reported by Duan et al. [23]. LAT1 expression was notably decreased in H_2_O_2_ treatment but restored by Ba. Although pretreatment with probiotic significantly decreased ASCT1 and ASCT2 expression, there was no significant change but a downward trend for GLUT2 mRNA expression in the Ba pretreatment group, which indicated that Ba treatment might restore the expression of those transporters. In the case of a single Ba group, ASCT1 and LAT1 expression was getting a reduction; furthermore, the result documented that in the pretreatment group EAAC1 mRNA expression was significantly increased. These phenomena may be related to the treatment time of probiotics, and the specific mechanism remains to be studied. Some other results showed that the mRNA expression of GLUT2 in probiotic-treated broilers was higher than control, but only numerically upregulated expression of SGLT1 and PepT-1 [24]. FAT/CD36 plays a role in the uptake of LCFA by small intestinal enterocytes [3]. The author reported that *Lactobacillus plantarum* LG42 could significantly decrease FAT/CD36 mRNA expression compared with untreated 3T3-L1 cells [25], but in our research, Ba failed to induce any effect on FAT/CD36 or PepT-1 expression, which is consistent with analyses by Even et al. [26].

Tight junctions (TJs) are the intercellular junctional complex that belong to a part of intestinal barrier; they prevent paracellular diffusion of microorganisms and other antigens across the epithelium [4], thus playing an important role in inflammatory bowel disease (IBD). Claudins, occludin, and zonula occludens (ZO) are common tight junctional proteins. In most cases, probiotics have shown enhanced effects on TJs in vivo and in vitro [27, 28]; our findings demonstrated the protective ability of Ba on occludin mRNA level, although Ba decreased the expression of ZO-1, H_2_O_2_ and Ba pretreatment group showed no effect on ZO-1. There are many evidences for the role of probiotics on TJs but a limited number of studies report the impact of oxidative stress on TJs. Catanzaro et al. showed that H_2_O_2_ caused severe disruption on ZO-1 and occludin expression in Caco-2 cells via immunostaining [29]. The underlying mechanism of our results may be that cells require more tight junctions under certain pathologic conditions.

In particular, oxidative stress-induced apoptosis most commonly occurs in GIT because of importance of normal function for integrated intestinal mucosa [5]. Apoptosis is recognized as a genetically controlled form of cell death and plays a crucial role in maintaining cellular balance; what’s more, some biochemical changes could occur in apoptotic cells such as protein cleavage, protein cross-linking, and DNA breakdown [30]. Caspases are pro-apoptotic proteins, which are widely expressed in most cells, amplifying the apoptotic signaling pathway and thus leading to rapid cell death. Moreover, Bcl-2 belongs to the Bcl-2 family that is regarded as an anti-apoptotic protein [31]. Probiotics have been identified and proposed to benefit apoptosis. Cui et al. have observed that supplementation with *Lactobacillus reuteri* ZJ617 helped attenuate LPS-induced gut apoptosis by decreasing caspase-3 activity [32]. Analogously, a probiotic strain named “USA-made” *VSL#3* exerted the protective action on H_2_O_2_-dependent ROS generation and cell damage on intestinal epithelial cells (IEC-6) [33]. Our data have shown a role for H_2_O_2_ in decreasing the expression of Bcl-2, and Ba pretreatment reversed this effect, indicating that this strain protected the cells from stress-induced apoptosis. However, H_2_O_2_-induced gene expression of caspase-3 was not inhibited by probiotic pretreatment. 

In conclusion, our results showed that probiotic Ba could protect porcine intestinal epithelia from oxidative stress damage through regulating absorptive transporters, tight junctions, and apoptosis gene expression. These effects indicated that Ba might make a preventative and economic contribution in treating oxidative stress diseases in animal husbandry. However, definite conclusions need to be verified by in vivo experiments and specific mechanisms.
